# Supplementary material for: An Immunoinformatics Approach for SARS-CoV-2 in Latam Populations and Multi-Epitope Vaccine Candidate Directed towards the World’s Population
Source: Vaccines (Basel). 2021 Jun 1;9(6):581. doi: 10.3390/vaccines9060581 (PMC8228945; doi:10.3390/vaccines9060581)
Supplement: Supplementary file 1 [file vaccines-09-00581-s001.zip › suppMat/supplementaryMaterial.pdf]

AN IMMUNO-INFORMATICS APPROACH FOR SARS-COV-2 IN LATAM POPULATIONS AND MULTI-EPITOPE VACCINE CANDIDATE DIRECTED TOWARDS THE WORLD'S POPULATION

Andrés F. Cuspoca MD
Grupo de Investigación en Epidemiología Clínica de Colombia (GRECO), Universidad Pedagógica y Tecnológica de Colombia, Colombia.
Telephone: +57 3215059664
ORCID: https://orcid.org/0000-0002-2420-4898

Laura L. Díaz
Grupo de Investigación en Epidemiología Clínica de Colombia (GRECO), Universidad Pedagógica y Tecnológica de Colombia, Colombia.
Telephone: +57 3209463160
ORCID: https://orcid.org/0000-0001-9030-6043

Alvaro F. Acosta

Grupo de Investigación en Epidemiología Clínica de Colombia (GRECO), Universidad Pedagógica y Tecnológica de Colombia, Colombia.
Telephone: +57 3222394899
ORCID: https://orcid.org/0000-0003-0849-8248

Marcela K. Peñaloza
Grupo de Investigación en Epidemiología Clínica de Colombia (GRECO), Universidad Pedagógica y Tecnológica de Colombia, Colombia.
Telephone: +57 3183204023
ORCID: https://orcid.org/0000-0002-2736-0538

Yardany R. Mendez MD

Grupo de Investigación en Epidemiología Clínica de Colombia (GRECO), Universidad Pedagógica y Tecnológica de Colombia, Colombia.

Telephone: +57 300 6004649
ORCID: https://orcid.org/0000-0003-1528-2672

Diana C. Clavijo
Pontificia Universidad Javeriana Cali, Facultad de Ingeniería y ciencias, Colombia
Telephone: +57 301 5844932
ORCID: https://orcid.org/0000-0003-0941-4552

Juvenal Yosa Reyes†
Universidad Simón Bolivar, Facultad de Ciencias Básicas y Biomédicas, Laboratorio de Simulación Molecular, Colombia
Telephone: +57 301 5844932 E-mail: juvenal.yosa@unisimonbolivar.edu.co
ORCID: https://orcid.org/0000-0002-6492-2640

*These authors contributed equally to this work.

†Corresponding Author.

Supplementary figures

Figure 1. SARS-CoV-2 Genome Structure.

The genome size varies from 29.8 kb to 29.9 kb, the 5' more than two-thirds of the genome comprises orf1ab encoding orf1ab polyproteins, while the 3' one third consists of genes encoding structural proteins SP, E, MG, NP and accessory proteins.

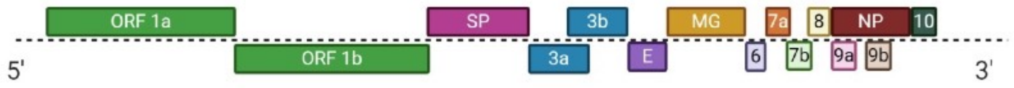

Figure 2: Development of vaccines against SARS-CoV-2

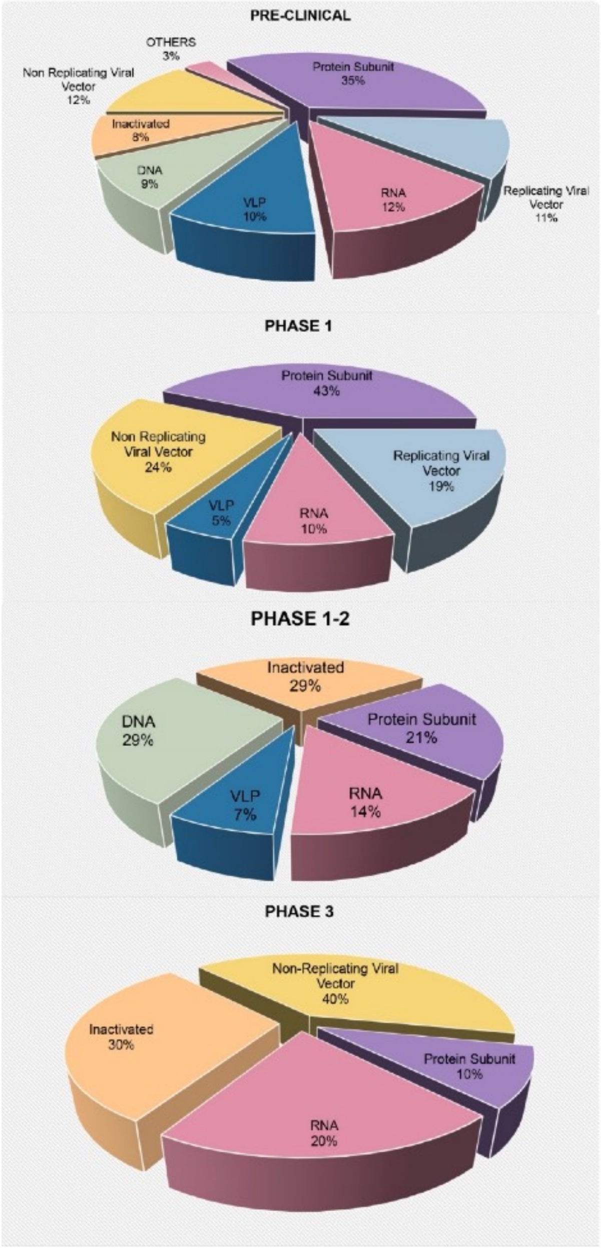

Figure 3. Innate immune response and damage of the signaling pathways by coronavirus proteins.

Once the virus enters the cell, the viral RNA is recognized by the TLR7, TLR3, RIG-I, and MDA5 receptors 1,2 activating the following signaling cascades: 1) translocation of nuclear factor kappa B (NF-kB) by TLR-7, to produce pro-inflammatory cytokines such as interleukins (IL) IL-1, IL-6 and tumor necrosis factor (TNF); 2) through TLR3, RIG-I and MDA5, nuclear translocation of IRF3 and IRF7 promote the synthesis IFN-I 3; and 3) activation of JAK-STAT and IRF9 by autocrine action of INF, promotes transcription of INF-stimulated genes 4. The proteins involved in the alteration of IFN-I production are nsp1, 3a, 3b, and 6. They alter the IFN-I production by inhibiting the nuclear translocation of STAT1. The nsp16 protein alters the production of IFN-I by preventing the detection of viral RNA by MDA5 5. The Papain-like protease protein (PL-PRO) antagonizes the production of IFN-I by inhibiting the phosphorylation of IRF3 6 while MP and NP alter the translocation of IRF3 and IRF7 7,8. Another innate altered immune response is the production of proinflammatory cytokines such as elevated IL-6 production, which is related to the action of the NP protein, which drives the translocation of NF-kB at the nuclear level 9,10. The "cytokine storm" could be associated with the expression of viral proteins such as 3a, 3b, and 7 11,12 that activate signaling pathways for the production of cytokines such as IL-8, CCL5, and TNF. The SARS-CoV envelope protein (E) is associated with inflammasome activation, which is related to lung damage[12].

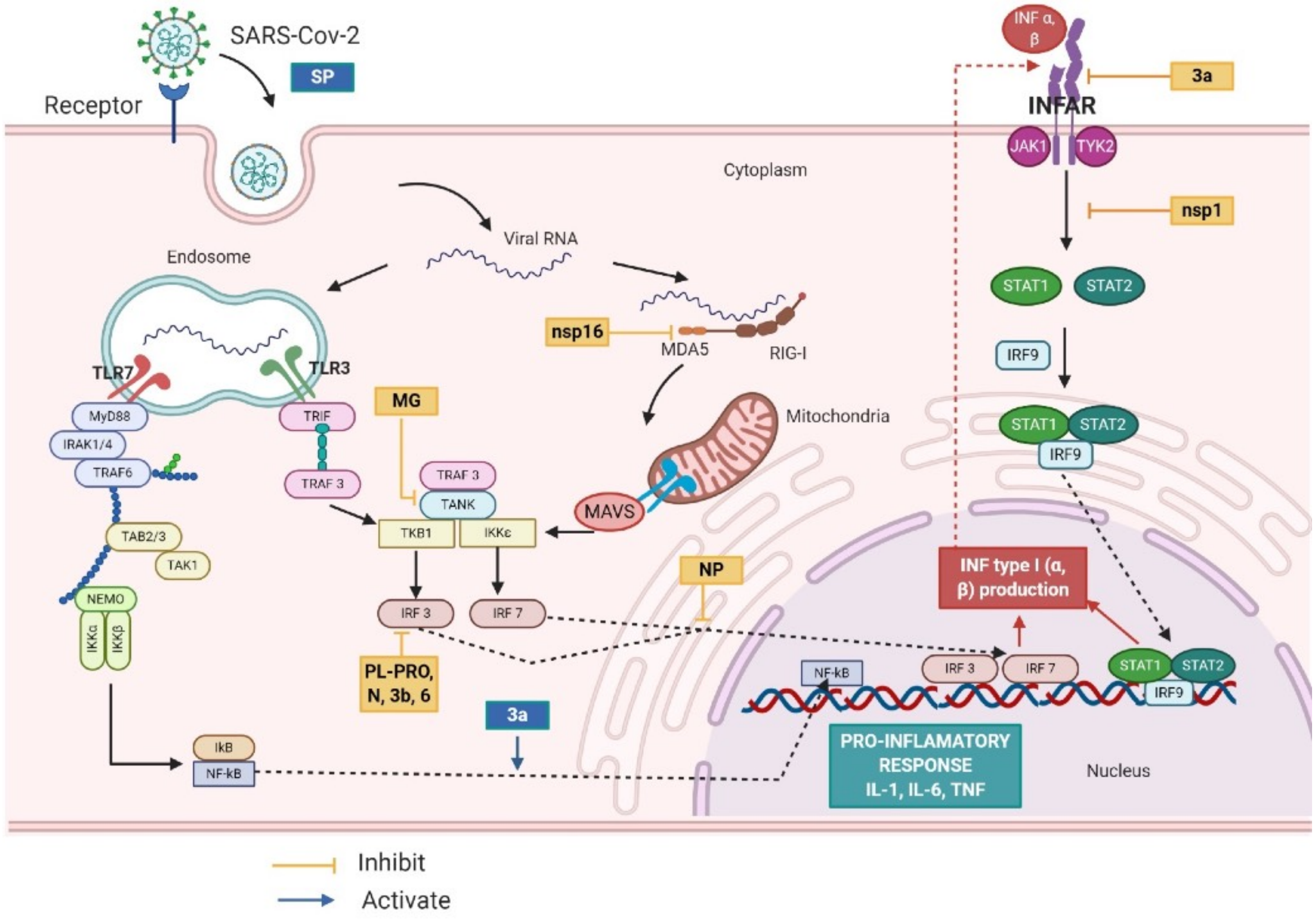

Figure 4: HLA-I by country: allelic frequency <0.1 are grouped in "others"

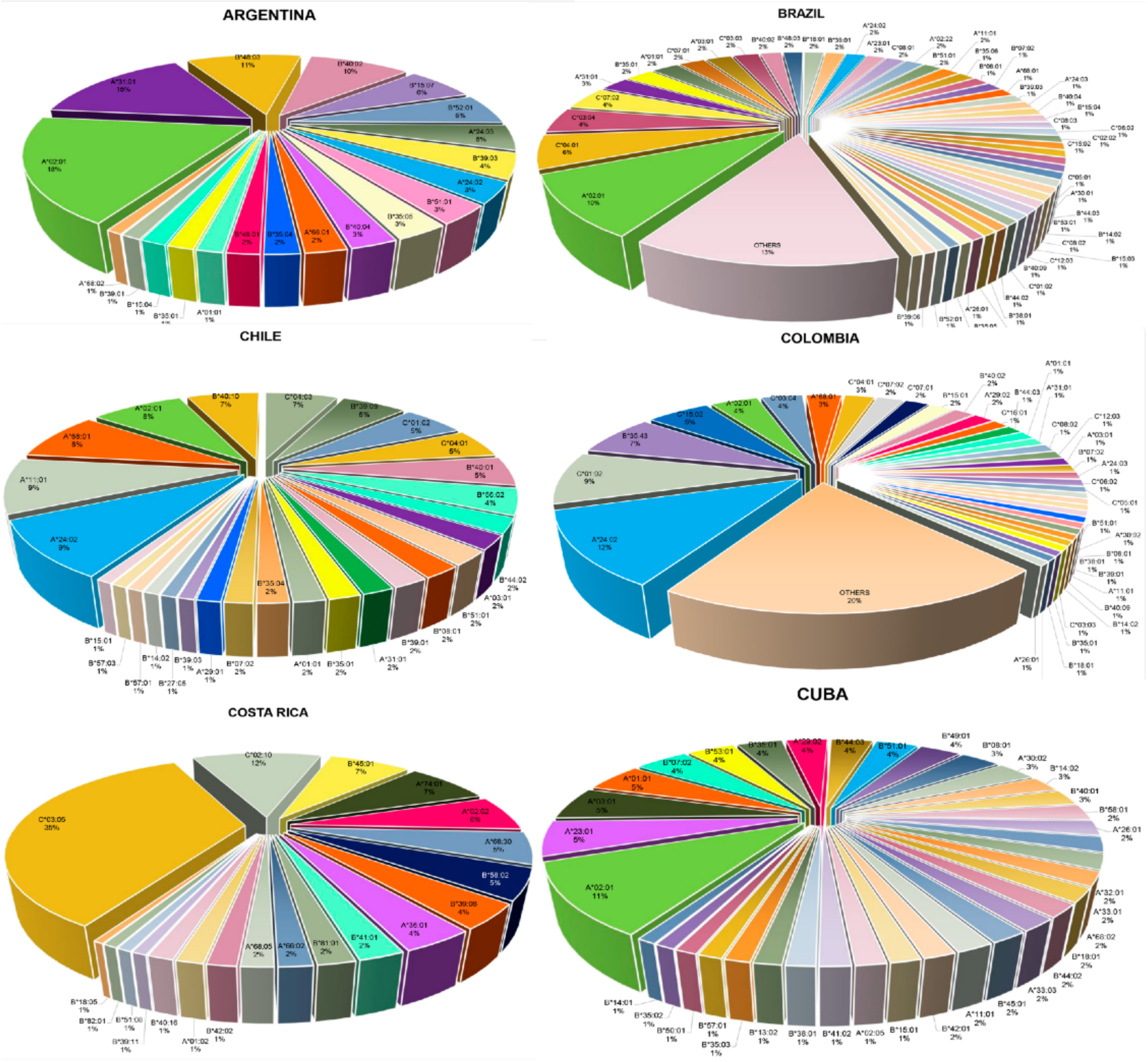

ECUADOR

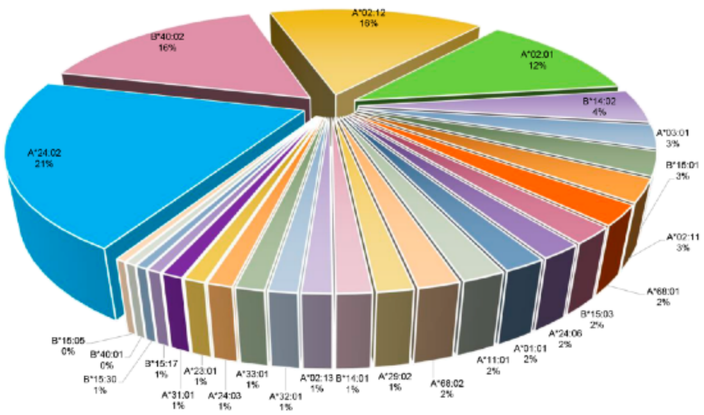

GUATEMALA

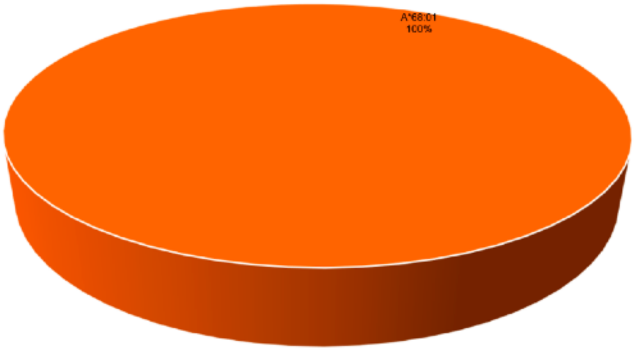

MARTINICA

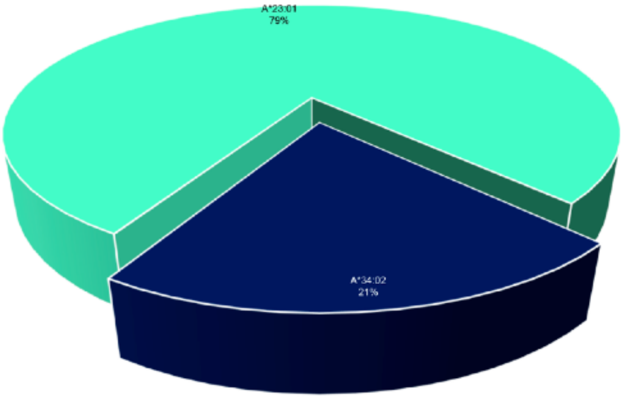

NICARAGUA

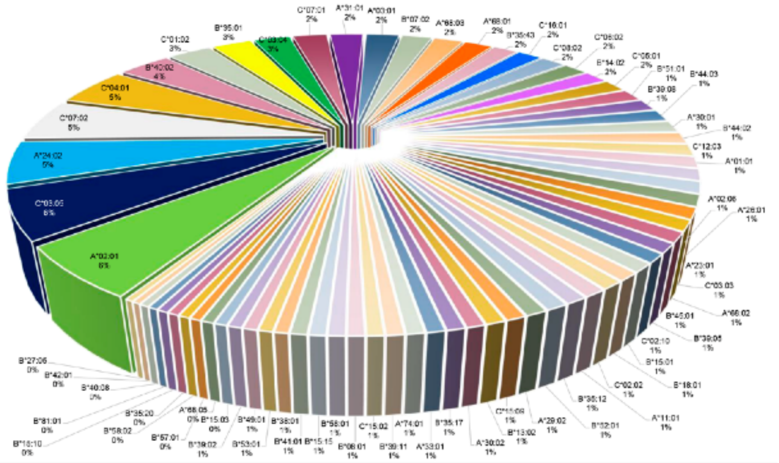

PERU

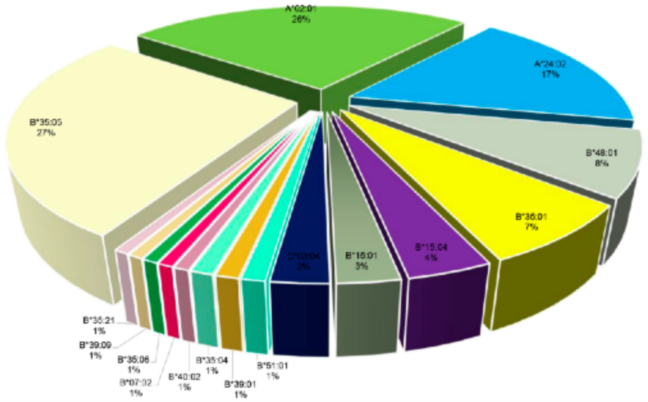

VENEZUELA

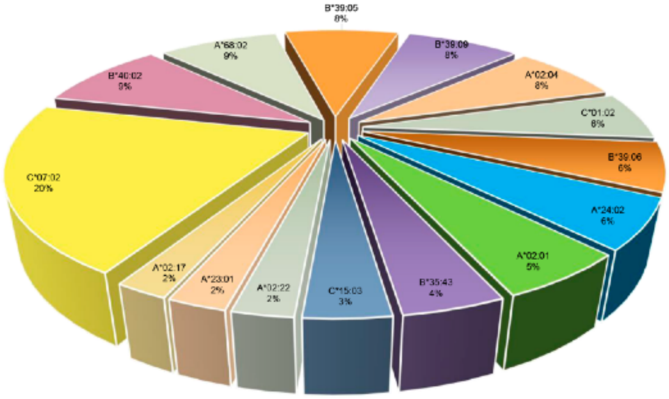

Figure 5: HLA-II by country.

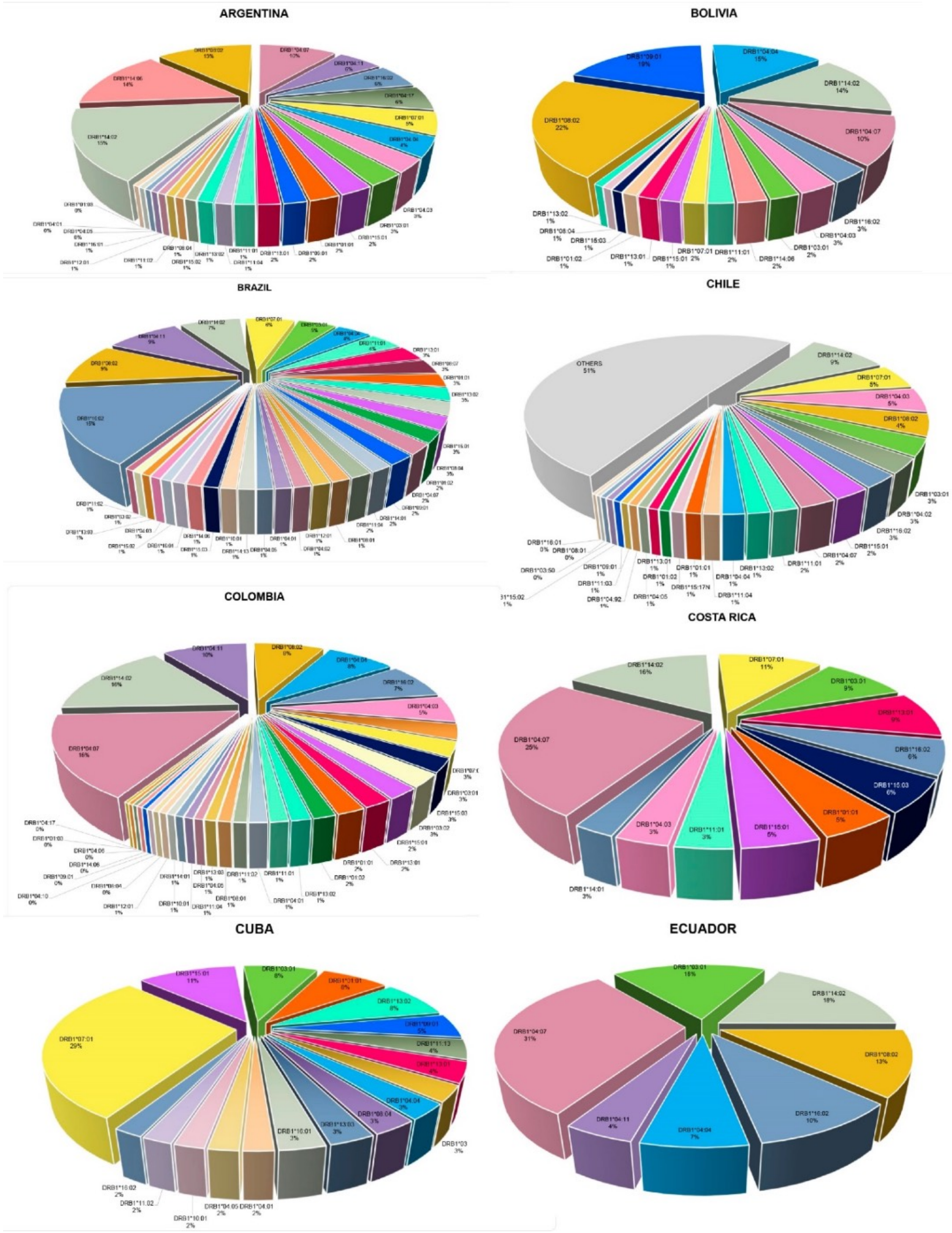

GUATEMALA

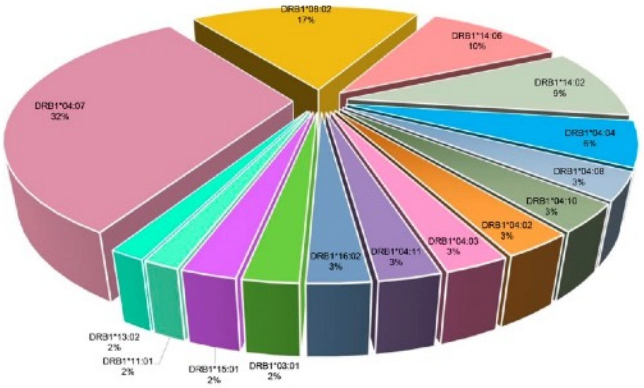

JAMAICA

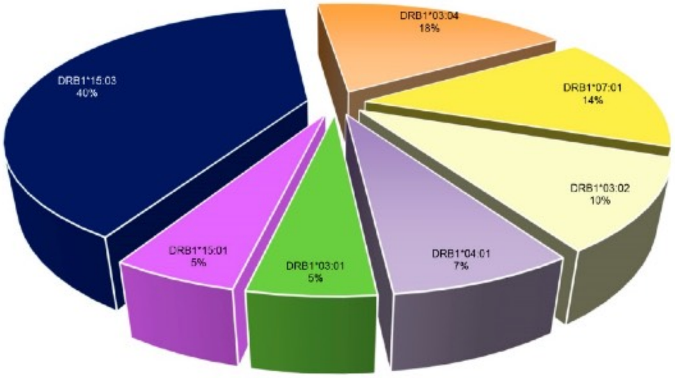

MARTINICA

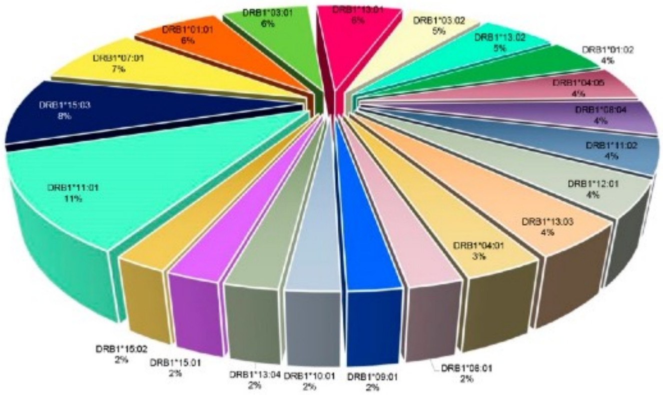

MEXICO

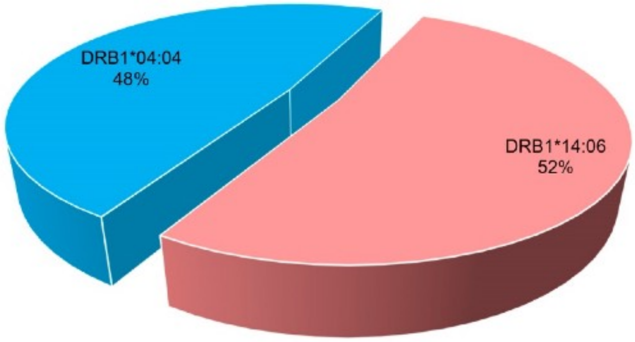

NICARAGUA

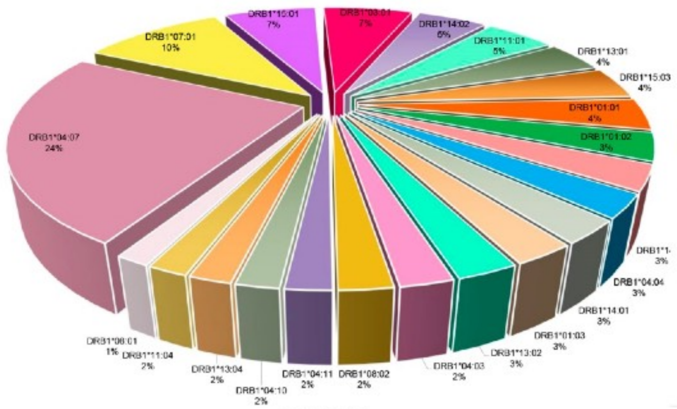

PANAMA

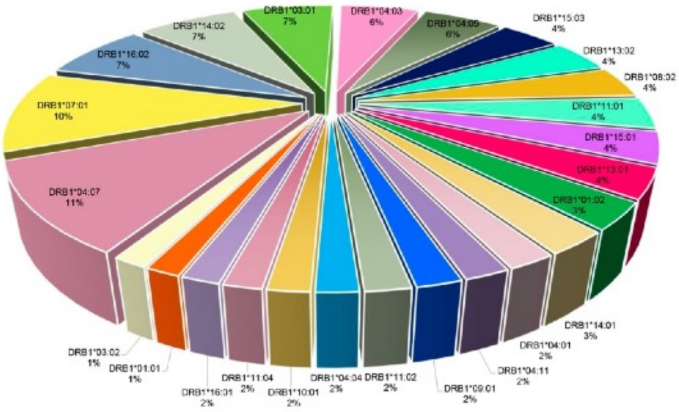

PARAGUAY

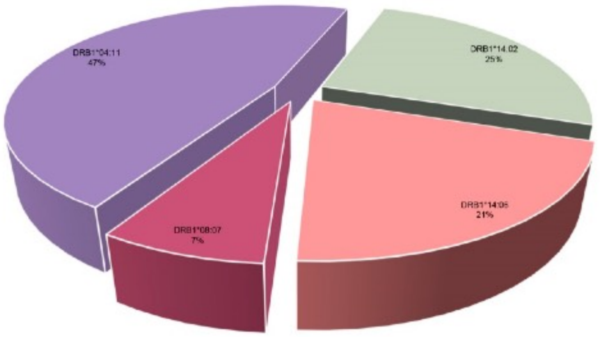

VENEZUELA

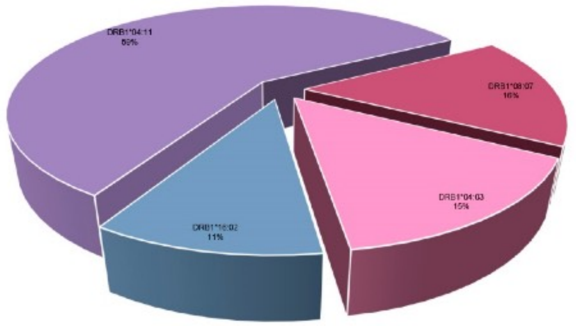

Figure 6: B cell epitopes zones.

a.) Ribbon diagram of RNA binding domain in NP b.) Surface structure RNA binding domain in NP with linear B cell epitopes in red, yellow, orange, green, and aquamarine that continue with opaque blue and light blue colors c.) Ribbon diagram of SP obtained from PDB:6M3M with the presence of Nglycosylations d.) Front view of SP with RBD domain in purple e.) Front view of SP with B cell epitopes in the lower region of the RBD domain f.) Lower view of SP, with discontinues epitopes in blue and linear epitopes in red, yellow, and orange.

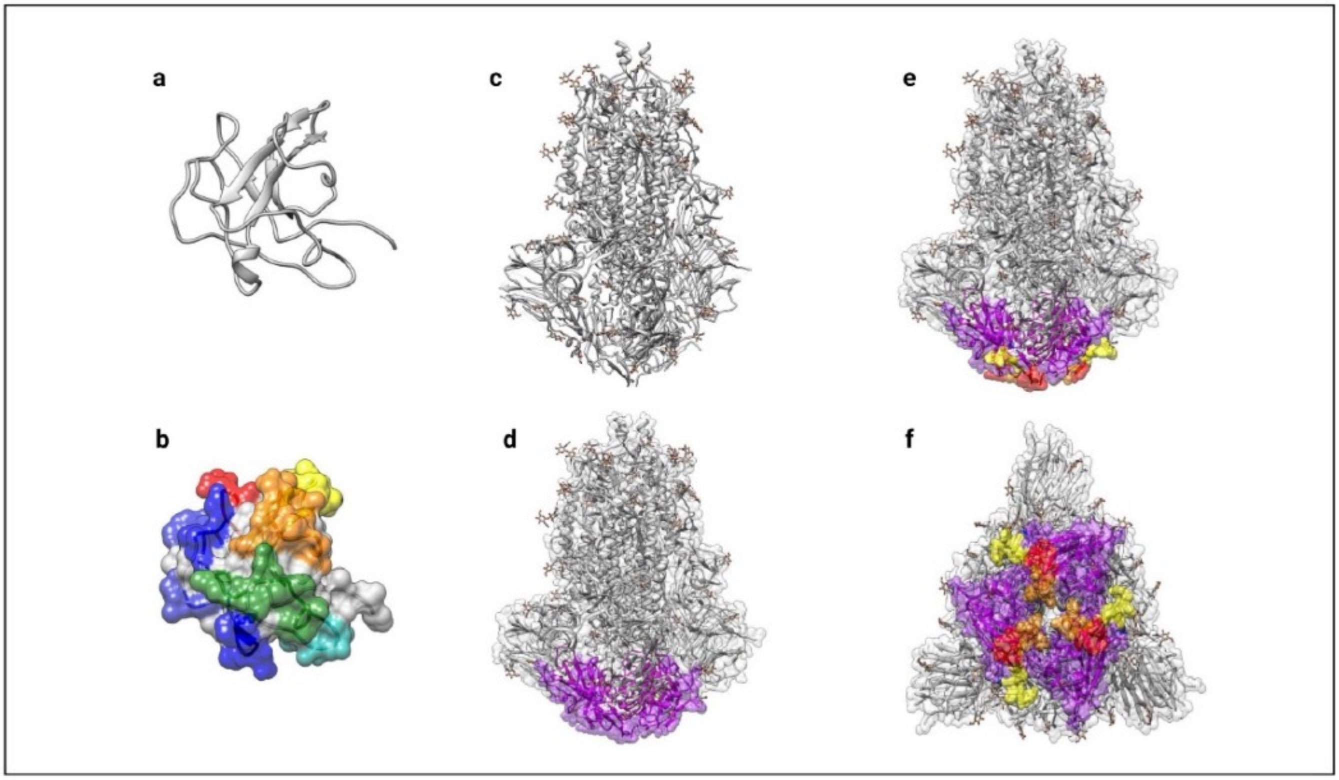

Figure 7: Prediction of the secondary structure of the multi-epitope construct from conserved regions of 92 SARS-CoV-2 proteomes.

a.) The sequence of vaccine construction along with the predicted secondary structure b.) The overall percentage of various secondary structures in vaccine construction as predicted by the SOPMA server c.) Representations of various secondary structures in the construction of the multi-epitope vaccine d.)The propensity for the appearance of various secondary structures according to the residues in the vaccine construction.

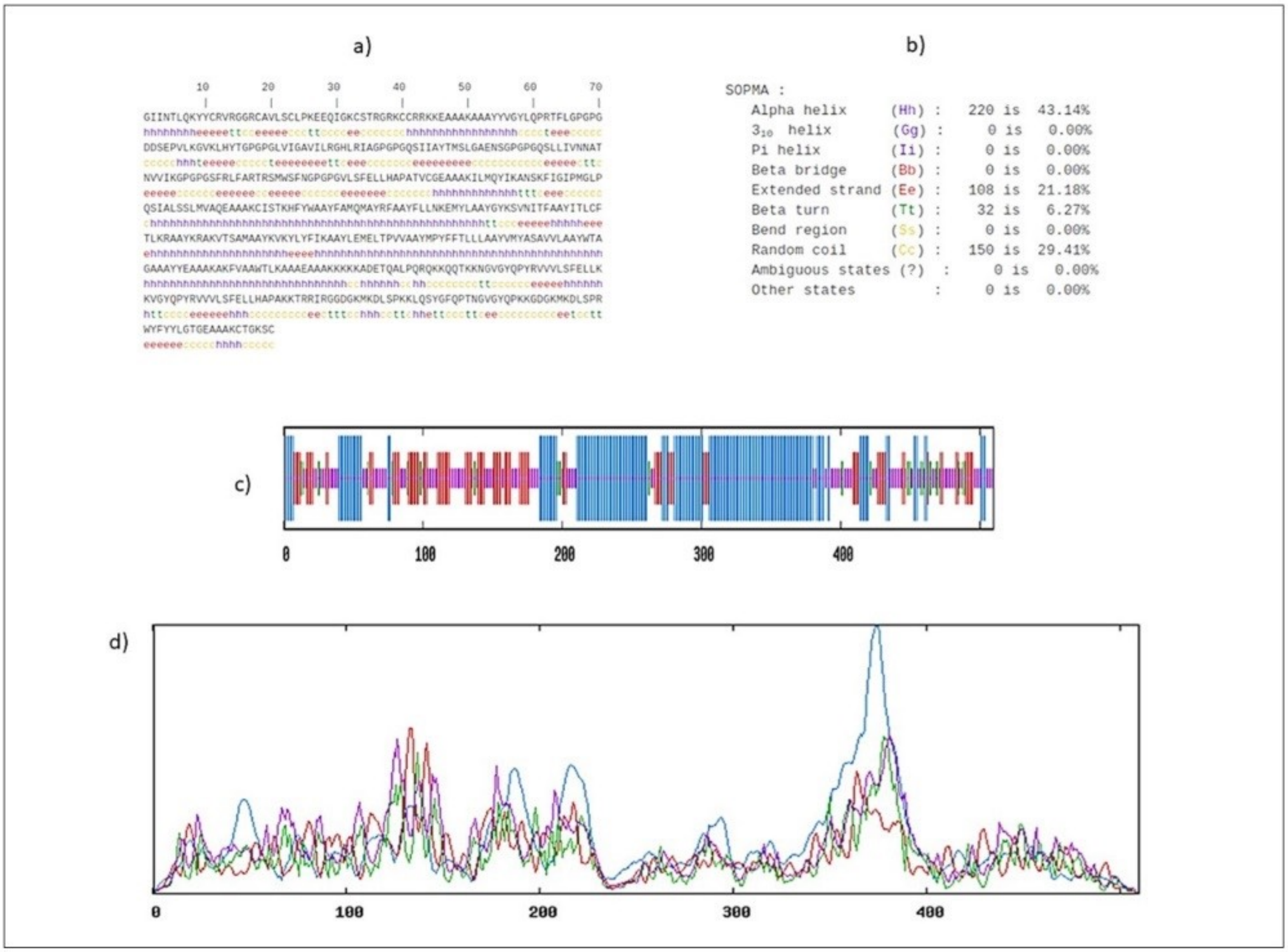

Figure 8: Secondary structure prediction

Structure prediction of the multi-epitope construct with PSIPRED 4.0

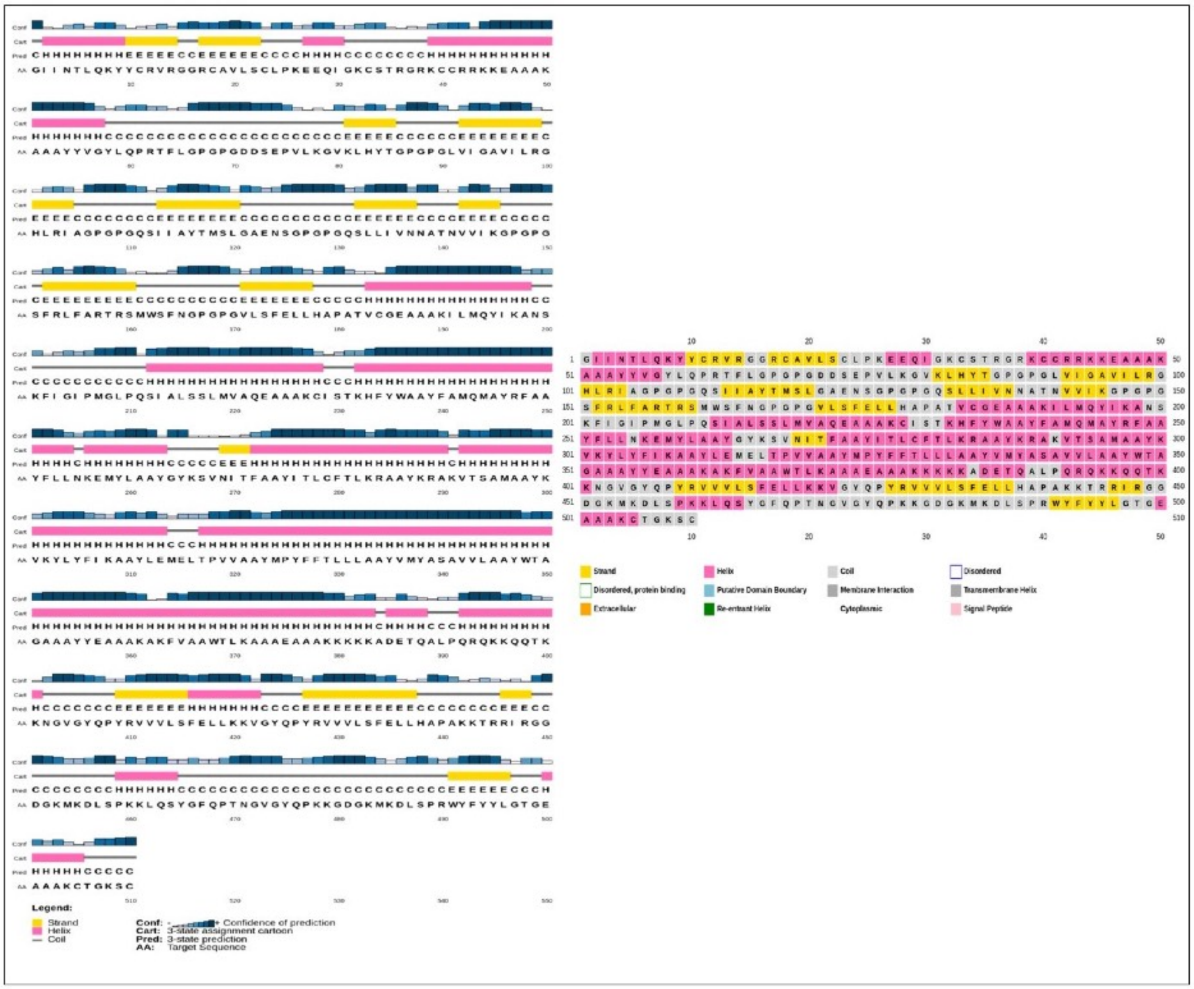

Figure 9: **Characteristics of the 3D structure of the multi-epitope construct.**

The tertiary structure of the vaccine was predicted by a thread-based homology model using the Robetta server, plotted in Pymol. The magenta, yellow, and red regions represent the set of epitopes of HTL, CTL, and BL respectively. a.) Analysis of the Ramachandran diagram obtained using PDBsum of the modeled structure revealed the presence of favorable regions of 88.7%, and 10.8% in the additionally allowed regions b.) The quality factor was analyzed using the ERRAT server and a quality factor of 90.78 that represents a good modeled structure was obtained c.) To refine the structure obtained, 3D refine and GalaxyRefine were used which resulted in an overall improvement given by the Ramachandran diagram of 91.5% in the most favored regions and 8.3% in the permitted regions d.) and by an ERRAT quality factor of 94.77.

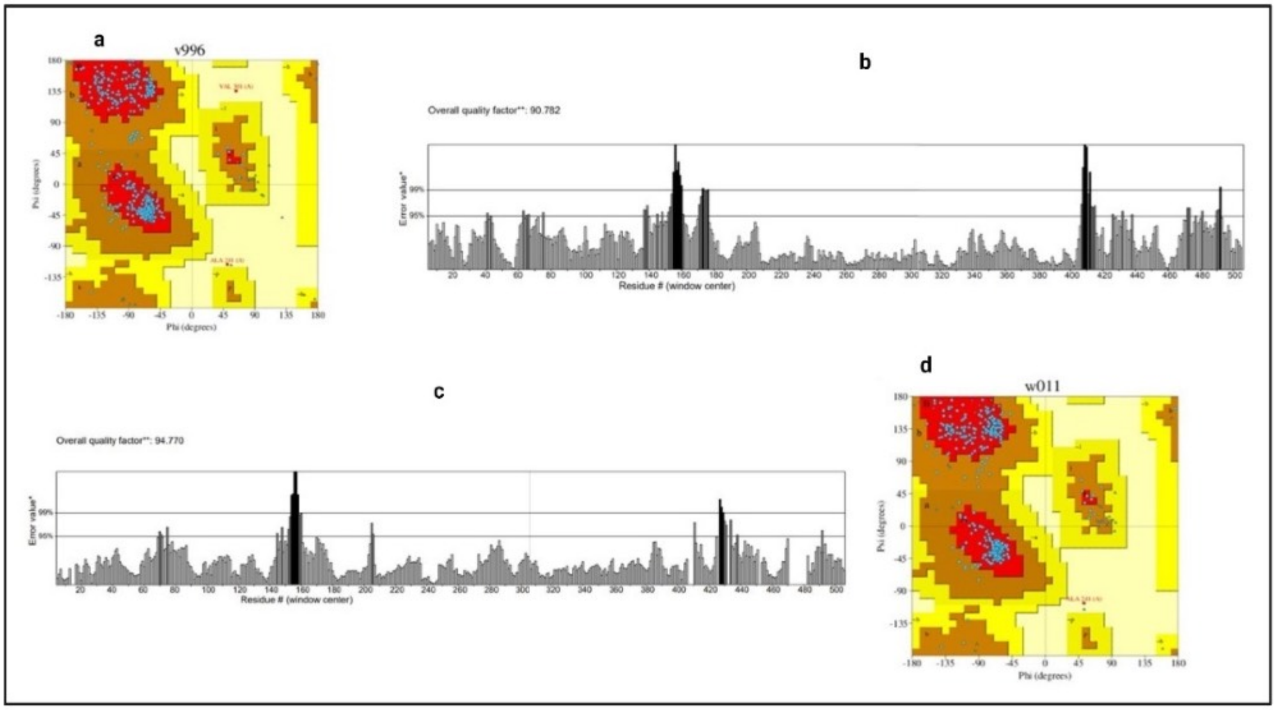

Figure 10: Molecular docking between the multiepitope construct and the TLR-4 / MD-2 heterodimer.

a.) Pose with lower energy obtained from the protein-protein molecular coupling obtained from ClusPro server v 2.0. The multi-epitope construct is in a position close to the TLR4 ectodomain and contacts MD-2 at the bottom when positioned in the concave cavity. b.) front view of MD-2 c.) back view of MD-2.

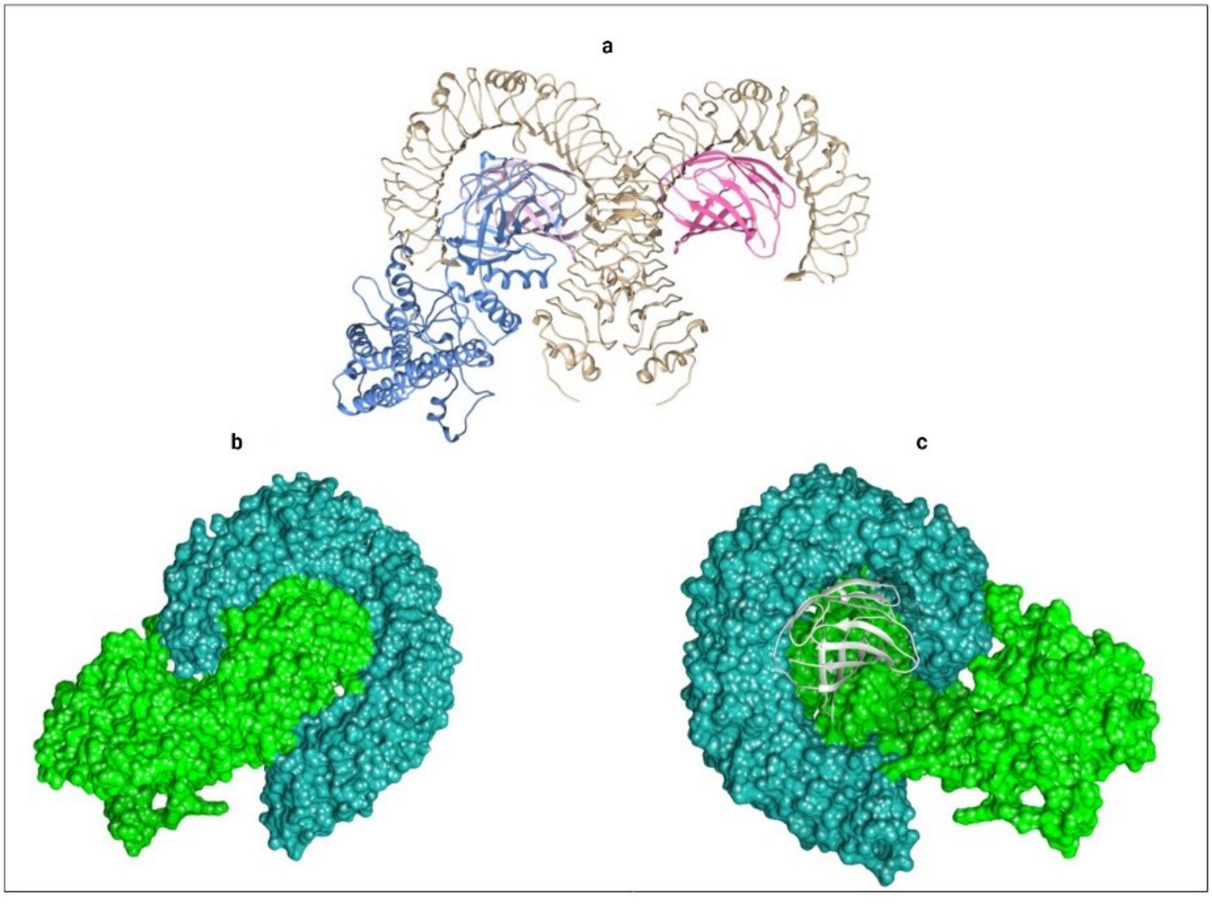

Figure 11: Intermolecular forces involving the molecular docking interface, between the multiepitope construct and TLR-4.

Hydrogen and salt bridges between the multiepitope construct and TLR4 a.) Hydrogen bridges (blue) b.) 2d diagram from PDB sum, Chain B TLR-4, Chain E multiepitope construct c.) Salt bridges (orange).

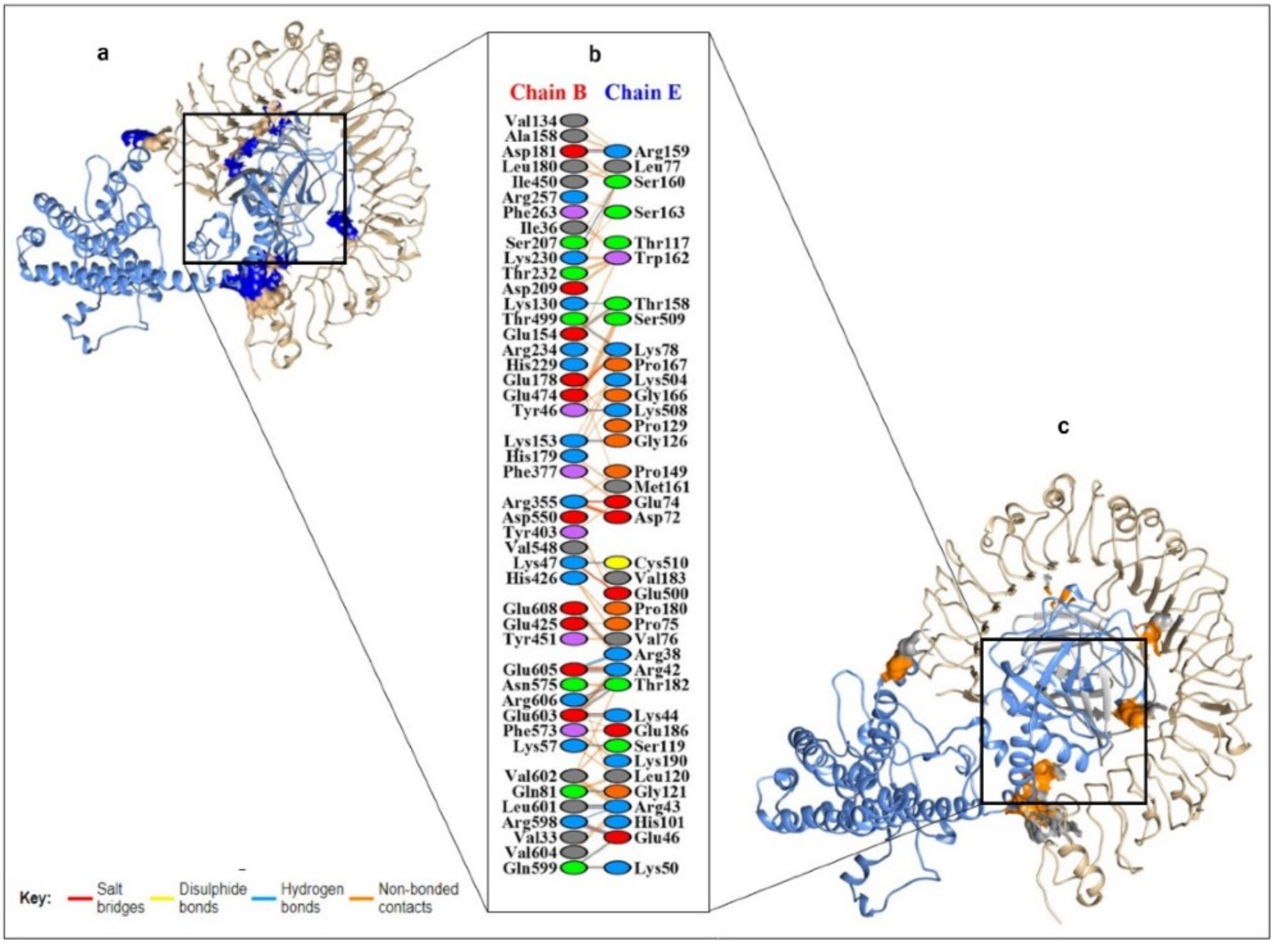

Figure 12: Intermolecular forces involving the molecular docking interface, between the multiepitope construct and MD-2.

hydrogen and salt bridges between the multiepitope construct and MD-2 a.) Hydrogen bridges (blue) b.) 2d diagram from PDB sum, Chain D MD-2, Chain E multi-epitope construct c.) Salt bridges (orange).

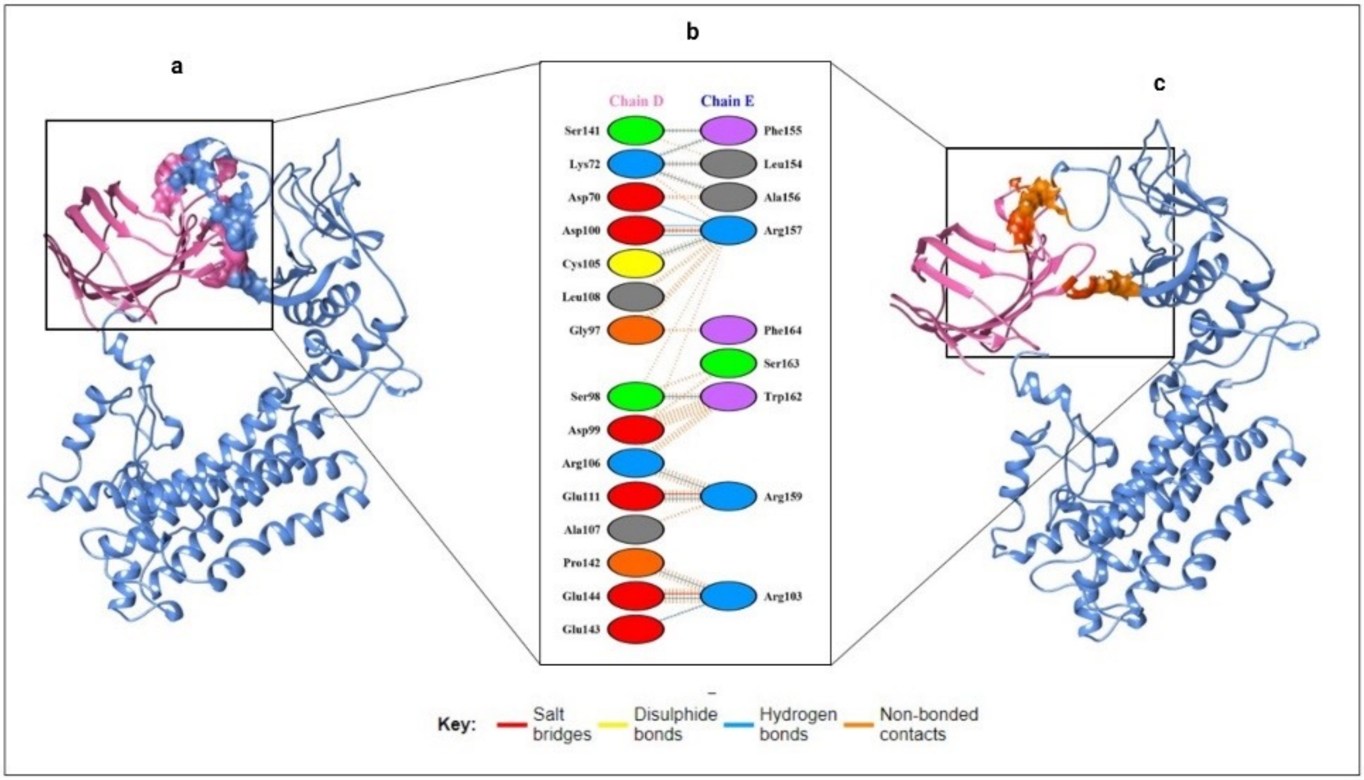

Figure 13: Salt bridges formation between TLR-4 and multi-epitope vaccine.

a.) a zoomed ball-and-stick representation of salt bridges. b.) Time series of salt bridges formed by the residue ASP-1424:ARG1639, ASP-756:ARG-1641, GLU-1180:ARG-1524, GLU-1183:ARG1520, and GLU-1556:ARG930

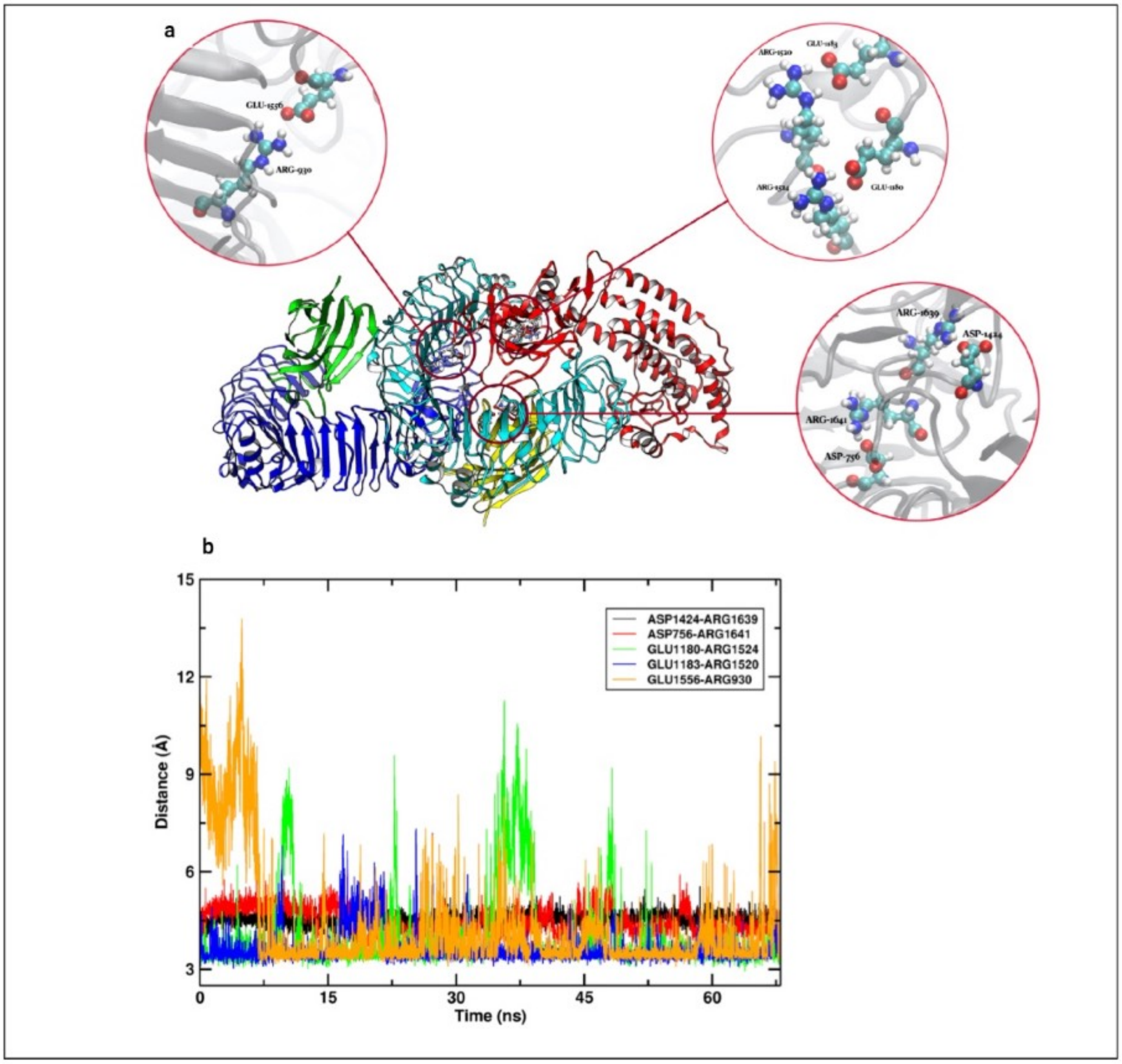

Figure 14: In silico cloning of the multi-epitope construct.

a.) The reverse translation used for optimization was performed using EMBOSS Backtranseq b.) Codon adaptation index value (CAI) of the cDNA before adaptation was observed to be 0.5690 with a GC content of 57.84% c.) The CAI score of the improved sequence was increased to 0.977 with a GC content of 50.71%. The improved CAI score represents the presence of the most abundant codons in Escherichia coli K12. d.) The adapted cDNA sequence was used for in-silico cloning purposes.

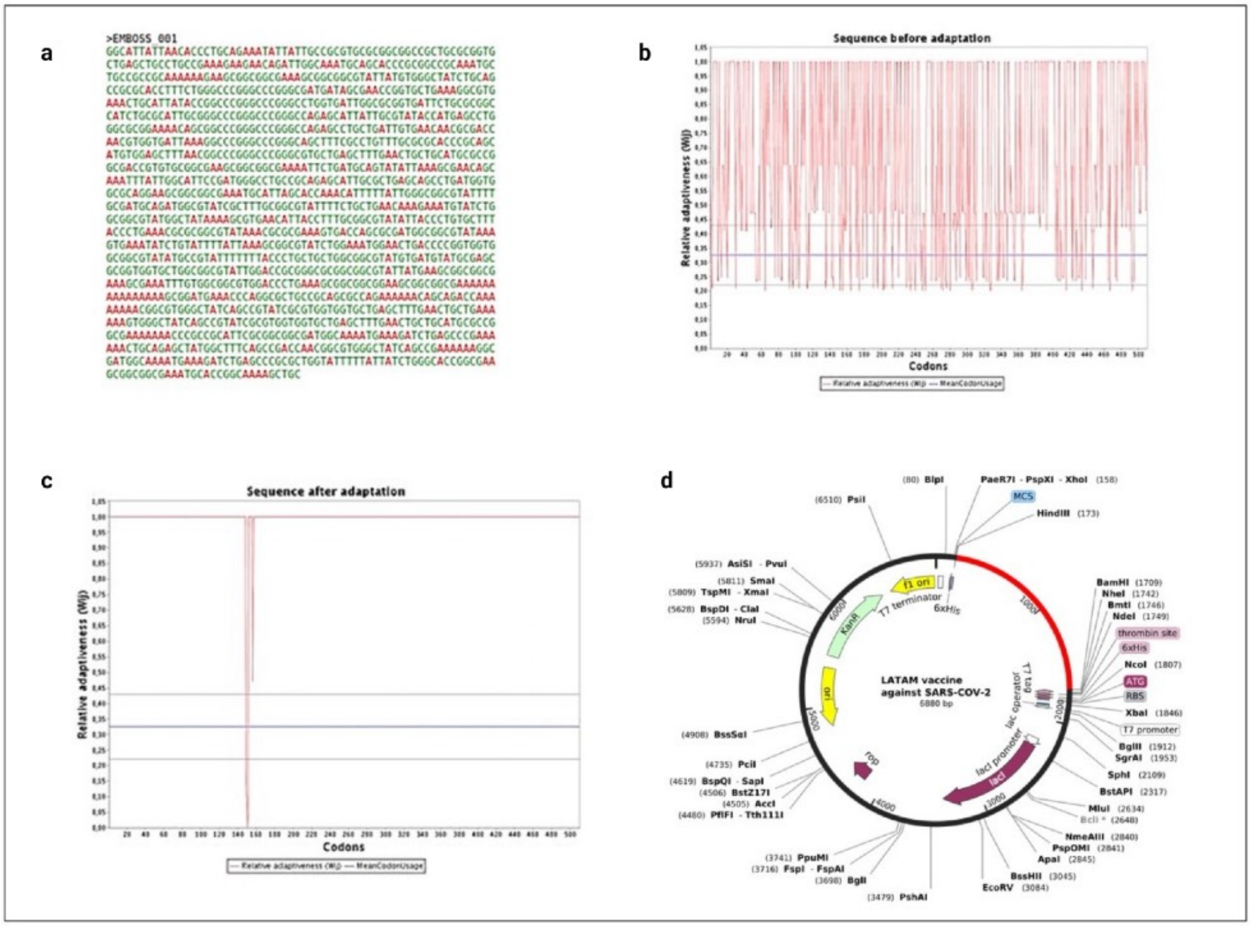

Figure 15: Extend immune simulation

a.) Total B-cell population and breakdown into IgG1-2 and IgM isotypes. As the time series progresses, a decrease in IgM and an increase in IgG is identified. This is consistent after day 311 of simulation and beyond day 460, but without antigenic stimulus. This suggests the presence of antibody-specific memory b.) CD4+ T-cell population. Memory persists beyond day 311 of simulation. This suggests the presence of CD4+ T memory beyond the antigen exposure that corresponds to the multi-epitope construct c.) Polarization towards a TH1 profile, which is related to the antigenic stimulus and is maintained during the simulation. This profile allows an adequate cellular interaction that mediates an adequate immune presentation d.) CD8+ T cell population, we see a constant stimulation of this cell lineage that is maintained beyond the period of stimulation by the vaccine construct e.) The population of antigen response-related cells, that are not professional presenters but secrete cytokines that stimulate other professional cells such as DC and MA. These are mediators of the adaptative immune response and are activated during the antigenic stimulus f.) The concentration of cytokines and interleukins that form a cellular response to the antigen. A greater amount of IFN γ and TGF-β is observed at the beginning of the exposure, as well as the presence of IL-2. In this graph, this is related to leukocyte growth.

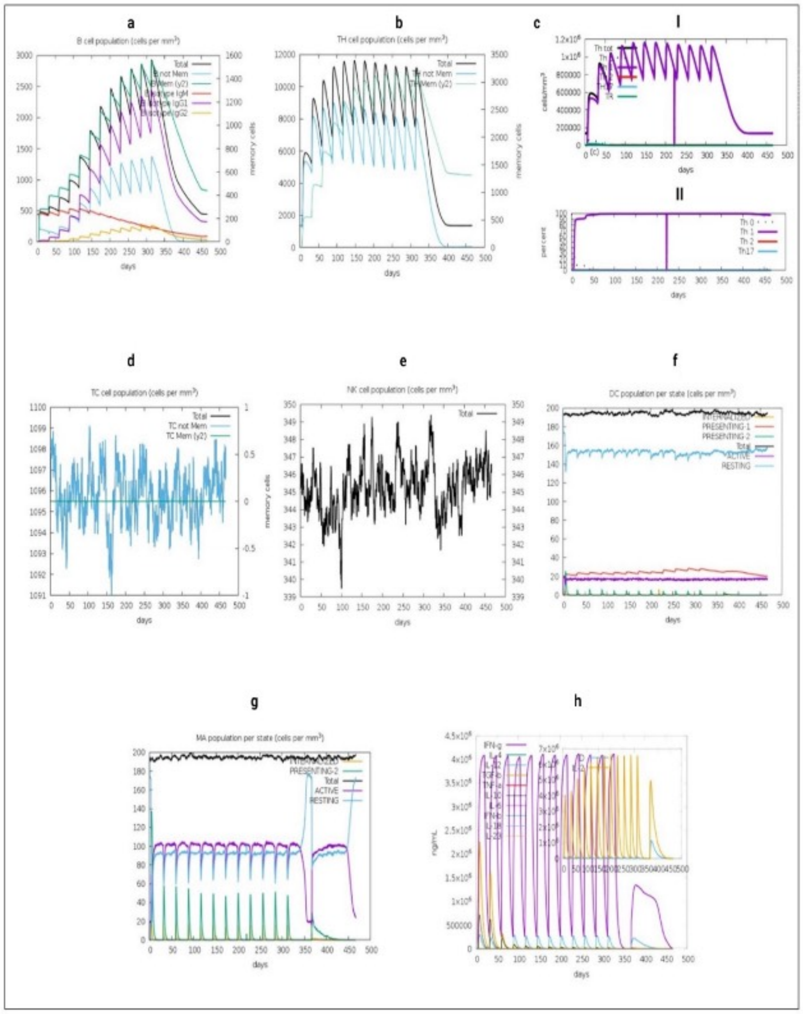

SUPPLEMENTARY REFERENCES:

1. Diebold, S. S., Kaisho, T., Hemmi, H., Akira, S. & e Sousa, C. R. Innate antiviral responses by means of TLR7-mediated recognition of single-stranded RNA. *Science (80-.).* 303, 1529–1531 (2004).

2. Kato, H. *et al.* Differential roles of MDA5 and RIG-I helicases in the recognition of RNA viruses. *Nature* 441, 101 (2006).

3. Li, G. *et al.* Coronavirus infections and immune responses. *J. Med. Virol.* 92, 424–432 (2020).

4. Prompetchara, E., Ketloy, C. & Palaga, T. Immune responses in COVID-19 and potential vaccines: Lessons learned from SARS and MERS epidemic. *Asian Pacific J. Allergy Immunol.* 38, 1–9 (2020).

5. Züst, R. *et al.* Ribose 2′-O-methylation provides a molecular signature for the distinction of self and non-self mRNA dependent on the RNA sensor Mda5. *Nat. Immunol.* 12, 137 (2011).

6. Devaraj, S. G. *et al.* Regulation of IRF-3-dependent innate immunity by the papain-like protease domain of the severe acute respiratory syndrome coronavirus. *J. Biol. Chem.* 282, 32208–32221 (2007).

7. Kopecky-Bromberg, S. A., Martínez-Sobrido, L., Frieman, M., Baric, R. A. & Palese, P. Severe acute respiratory syndrome coronavirus open reading frame (ORF) 3b, ORF 6, and nucleocapsid proteins function as interferon antagonists. *J. Virol.* 81, 548–557 (2007).

8. Siu, K.-L. *et al.* Severe acute respiratory syndrome coronavirus M protein inhibits type I interferon production by impeding the formation of TRAF3·TANK·TBK1/IKKϵ complex. *J. Biol. Chem.* 284, 16202–16209 (2009).

9. Zhang, X. *et al.* Nucleocapsid protein of SARS-CoV activates interleukin-6 expression through cellular transcription factor NF-κB. *Virology* 365, (2007).

10. LIAO, Q.-J. *et al.* Activation of NF-κB by the Full-length Nucleocapsid Protein of the SARS Coronavirus. *Acta Biochim. Biophys. Sin. (Shanghai).* 37, 607–612 (2005).

11. Kanzawa, N. *et al.* Augmentation of chemokine production by severe acute respiratory syndrome coronavirus 3a/X1 and 7a/X4 proteins through NF-κB activation. *FEBS Lett.* 580, 6807–6812 (2006).

12. Law, H. K. W. *et al.* Chemokine up-regulation in sars-coronavirus--infected, monocyte-derived human dendritic cells. *Blood* 106, 2366–2374 (2005).
